# Polymorphic Ventricular Tachycardia and Torsades in Methadone Therapy Captured by Loop Recorder

**DOI:** 10.1016/j.jaccas.2026.108796

**Published:** 2026-06-18

**Authors:** Resha Reya Ganthan, Ahmed Elcicek, Joud Fahed, Rayna Isber, Nidal Isber

**Affiliations:** a Department of Internal Medicine, Richmond University Medical Center, Staten Island, New York, USA; b Department of Internal Medicine, Ascension Saint Agnes Hospital, Baltimore, Maryland, USA; c Department of Biology, Barnard College, Manhattan, New York, USA; d Department of Electrophysiology, Richmond University Medical Center, Staten Island, New York, USA

**Keywords:** implantable cardioverter-defibrillator, implantable loop recorder, methadone, polymorphic ventricular tachycardia, torsades de pointes,, ventricular fibrillation

## Abstract

**Background:**

Methadone is associated with QT prolongation and risk of torsades de pointes, particularly at higher doses, and management becomes challenging when the precipitating factor cannot be eliminated.

**Case Summary:**

A 56-year-old man on high-dose methadone with an implantable loop recorder experienced 3 episodes of polymorphic ventricular tachycardia and ventricular fibrillation, including a prolonged 94-second syncopal event. Device electrograms demonstrated pause-dependent initiation with short–long–short sequences and marked QT prolongation preceding arrhythmia onset. Despite limited follow-up, he ultimately agreed to wearable cardioverter-defibrillator therapy and subsequent implantable cardioverter-defibrillator (ICD) placement for secondary prevention.

**Discussion:**

This case highlights methadone-associated malignant ventricular arrhythmias, the importance of continuous rhythm monitoring, and the role of ICD insertion when the offending agent cannot be safely withdrawn.

**Take-Home Message:**

In high-risk patients with recurrent ventricular arrhythmias on methadone, individualized monitoring and ICD therapy may be warranted.

## Visual Summary

**Figure:**
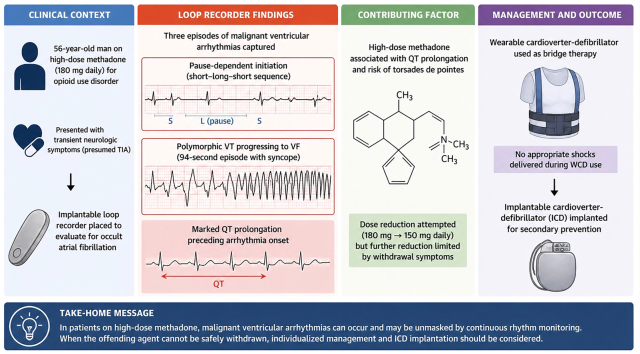

**Visual Summary:**
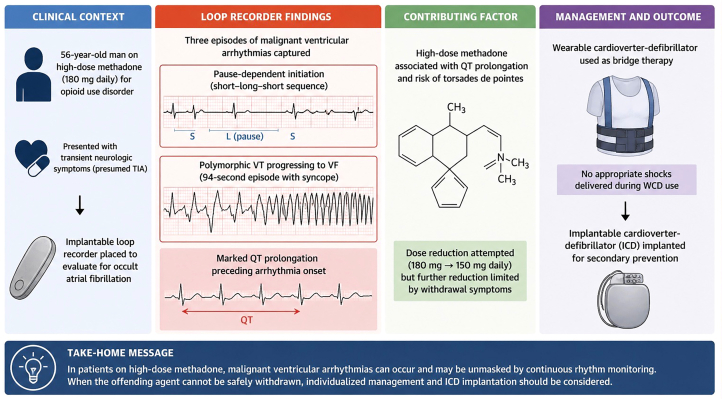
Methadone-Associated Polymorphic Ventricular Tachycardia Captured by Implantable Loop Recorder A patient receiving high-dose methadone developed recurrent pause-dependent polymorphic ventricular tachycardia (VT) consistent with torsades de pointes, progressing to ventricular fibrillation (VF). Continuous rhythm monitoring identified malignant arrhythmias despite limited tolerance to dose reduction. No appropriate shocks were delivered during wearable cardioverter-defibrillator use, and an implantable cardioverter-defibrillator was placed for secondary prevention. TIA = transient ischemic attack; WCD = wearable cardioverter-defibrillator.

Methadone is widely used for the treatment of opioid use disorder due to its long half-life and effectiveness in reducing withdrawal symptoms and cravings. However, methadone is associated with dose-dependent prolongation of the QT interval through inhibition of the rapid component of the delayed rectifier potassium current (IKr), predisposing patients to early afterdepolarizations and torsades de pointes.^1^^,^^13^ The risk of QT prolongation and malignant ventricular arrhythmias increases with higher methadone doses, particularly above 100 mg daily, although significant interindividual variability exists.^1^^,^^5^

**Take-Home Messages**

• High-dose methadone can precipitate pause-dependent torsades de pointes and ventricular fibrillation, particularly in patients with persistent QT prolongation.
• Continuous rhythm monitoring may identify clinically silent malignant arrhythmias and help guide secondary prevention ICD therapy when risk factors cannot be fully reversed.

Although the overall incidence of torsades de pointes in patients receiving methadone is relatively low, the consequences can be life-threatening, especially in the presence of additional risk factors such as electrolyte abnormalities, concomitant drug use, or poor follow-up.^3^^,^^4^^,^^7^ Current management strategies emphasize electrocardiographic monitoring and modification of reversible risk factors. However, in patients in whom methadone cannot be safely reduced or discontinued, optimal management remains uncertain.

We present a case of recurrent polymorphic ventricular tachycardia in a patient on high-dose methadone, captured by an implantable loop recorder, highlighting challenges in risk mitigation, follow-up adherence, and the role of implantable cardioverter-defibrillator (ICD) insertion for secondary prevention.

## Case Presentation

**Figure 1:**
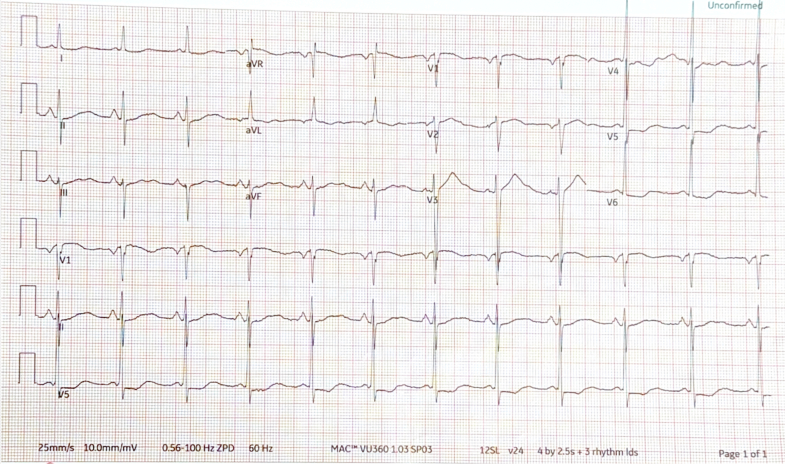
Baseline 12-Lead Electrocardiogram Demonstrating Sinus Rhythm With Marked Prolongation of Corrected QT Interval, Consistent with Increased Susceptibility to Torsades de Pointes

A 56-year-old man with a history of opioid use disorder on high-dose methadone maintenance therapy, prior transient ischemic attack/cerebrovascular accident, and an implantable loop recorder placed following a presumed transient ischemic attack to evaluate for occult atrial fibrillation as a potential cardioembolic source presented for evaluation of recurrent ventricular arrhythmias detected on remote monitoring. The patient demonstrated significant challenges with follow-up adherence, including failure to respond to multiple outreach attempts after initial arrhythmic events. A baseline 12-lead electrocardiogram demonstrated sinus rhythm with a prolonged corrected QT interval (QTc) of approximately 550 to 600 ms (Figure 1).

**Figure 2:**
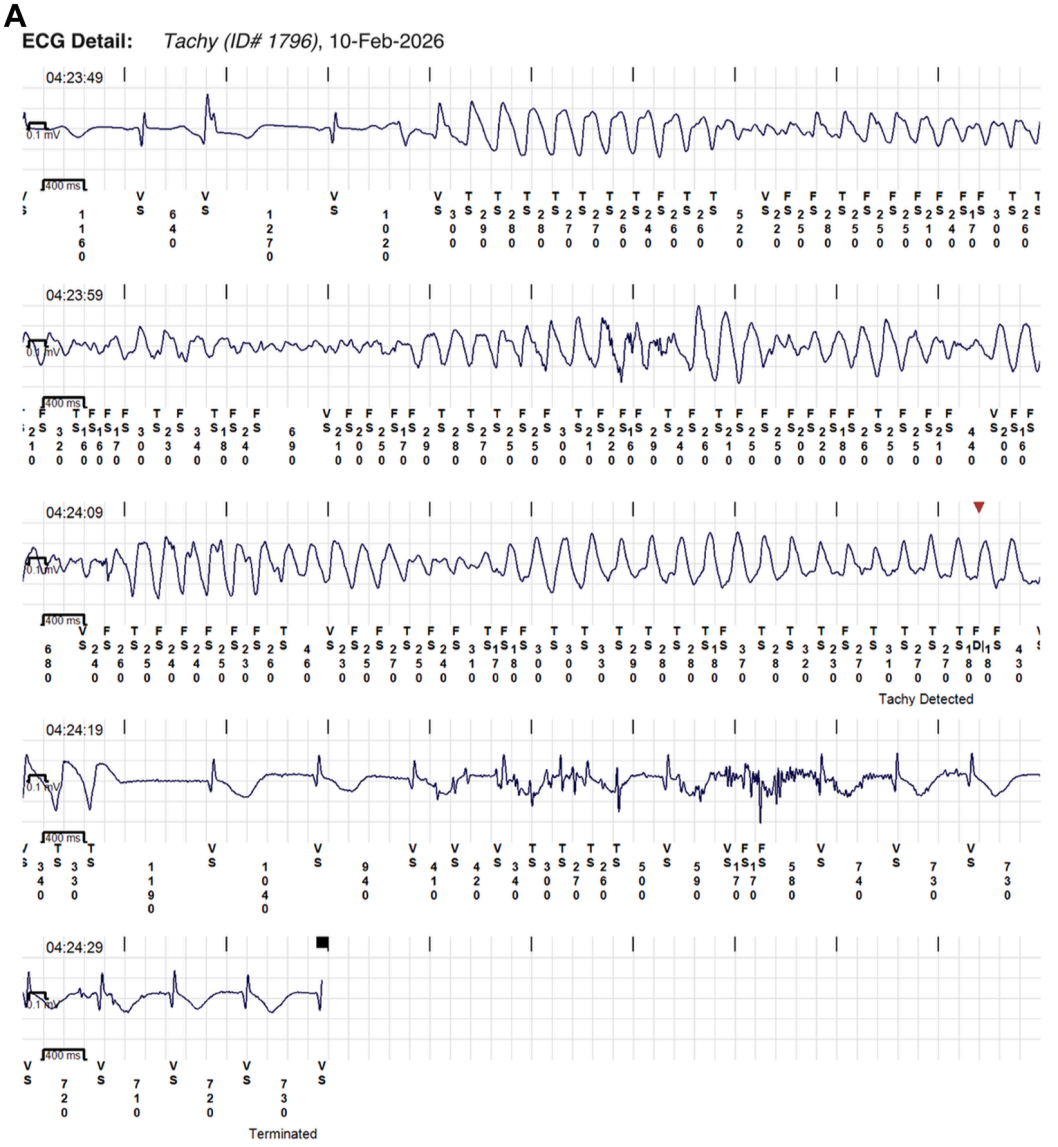

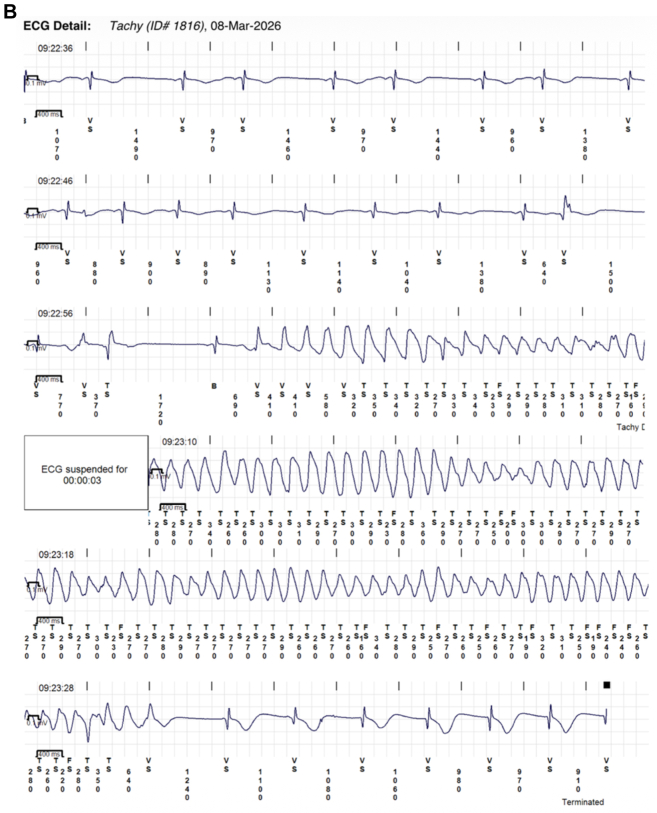

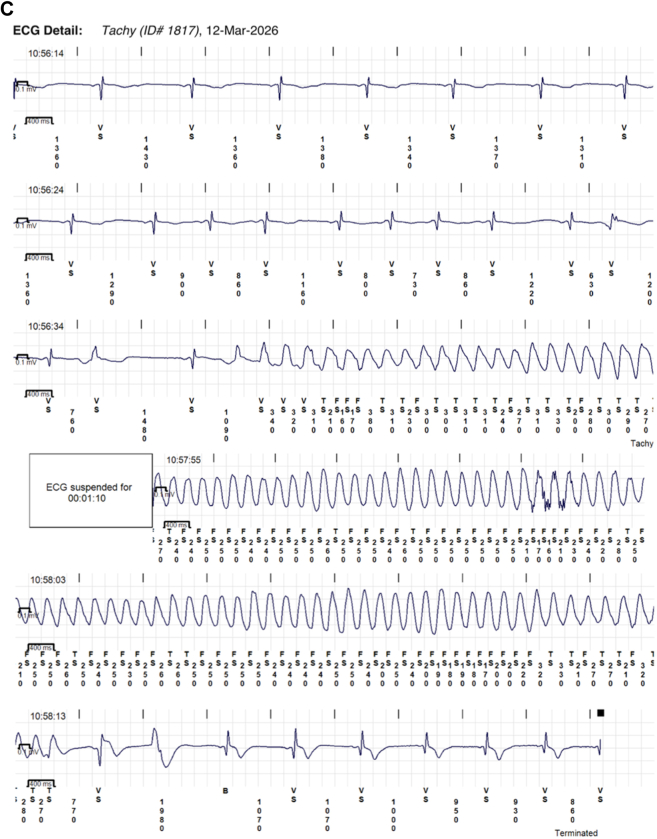
Device Electrograms Demonstrating Recurrent Polymorphic Ventricular Tachyarrhythmias (A) Episode 1 demonstrating initiation of polymorphic ventricular tachycardia during sinus rhythm with a short–long–short sequence, followed by torsades de pointes with beat-to-beat variation in morphology and spontaneous termination. (B) Episode 2 demonstrating recurrent polymorphic ventricular tachycardia with irregular cycle lengths and marked variability in amplitude and frequency. (C) Episode 3 demonstrating transition from polymorphic ventricular tachycardia into more disorganized ventricular fibrillation, followed by spontaneous termination. Collectively consistent with pause-dependent torsades de pointes in the setting of QT prolongation. ECG = electrocardiogram

The first documented episode occurred on February 10, 2026, at 4:24 am while the patient was asleep. Loop recorder interrogation revealed polymorphic ventricular tachycardia consistent with torsades de pointes at a rate of 222 beats/min, lasting 32 seconds, with spontaneous termination (Figure 2A). The episode began during sinus rhythm with ventricular cycle lengths of approximately 1,310 to 1,430 milliseconds, followed by a short–long–short initiation sequence and abrupt onset of polymorphic ventricular tachycardia. The arrhythmia demonstrated beat-to-beat variation in QRS morphology and amplitude, consistent with torsades de pointes, with an estimated ventricular cycle length of approximately 280 to 320 milliseconds. The preceding repolarization pattern suggested marked QT prolongation, with an estimated QT/QTc of approximately 600/650 milliseconds on the baseline rhythm strip. The patient was asymptomatic. Despite multiple attempts to contact him after the first event, including notification through law enforcement due to concern for his safety, he did not present for evaluation.

A second episode occurred on March 8, 2026, at approximately 9:20 to 9:22 am, consisting of polymorphic ventricular tachycardia/ventricular fibrillation at a rate of 231 beats/min, lasting 27 to 30 seconds, again terminating spontaneously (Figure 2B). This episode similarly began during sinus rhythm with ventricular cycle lengths of approximately 1,020 to 1,270 milliseconds, followed by ventricular ectopy and degeneration into highly irregular polymorphic ventricular tachycardia. The arrhythmia demonstrated marked variability in amplitude and cycle length (approximately 240-340 milliseconds), consistent with dynamic repolarization instability. Baseline rhythm again suggested significant QT prolongation, with estimated QT/QTc values in the range of 580 to 620/630 to 680 milliseconds. The patient reported dizziness, shortness of breath, flushing, and severe malaise, with probable loss of consciousness. He acknowledged intermittent heroin use and believed heroin may have been used around the time of this episode. He subsequently presented for evaluation, during which his methadone dose was reduced from 180 mg to 150 mg daily; however, further reduction was not tolerated due to significant withdrawal symptoms.

The third and most severe episode occurred on March 12, 2026, at 10:56 am while the patient was at rest, during which, the patient experienced marked dizziness and loss of consciousness. Loop recorder interrogation demonstrated ventricular fibrillation at a rate of 250 beats/min, lasting 1 minute and 34 seconds, with spontaneous termination (Figure 2C). The tracing demonstrated transition from sinus rhythm with premature ventricular activity into sustained high-rate polymorphic ventricular tachycardia with intermittent periods of relative organization, with ventricular cycle lengths of approximately 260 to 300 milliseconds before degeneration into more disorganized activity. The initiation pattern, morphology, and preceding pause-dependent sequence were most consistent with torsades de pointes degenerating into ventricular fibrillation in the setting of marked QT prolongation.

His medications included methadone 150 mg daily, which was continued as maintenance therapy for opioid use disorder, atorvastatin 80 mg daily, and magnesium supplementation. He also demonstrated difficulty maintaining follow-up. In the absence of detected atrial fibrillation and in light of subsequent malignant ventricular arrhythmias, the patient's initial neurologic symptoms—originally interpreted as a transient ischemic attack—may have represented transient arrhythmic events rather than true ischemia.

Given recurrent malignant ventricular arrhythmias, including torsades de pointes and prolonged ventricular fibrillation with syncope, the patient was deemed at high risk for sudden cardiac death. He initially refused hospitalization and ICD insertion but, following the third event, agreed to a wearable cardioverter-defibrillator as a bridge to ICD insertion for secondary prevention.

Overall, device-recorded electrograms demonstrated pause-dependent polymorphic ventricular tachycardia/torsades de pointes characterized by long sinus cycle lengths, short–long–short initiation sequences, marked QT prolongation, and spontaneous termination, supporting a mechanism of methadone-associated triggered ventricular arrhythmia.

## Discussion

This case illustrates the complex interplay between methadone therapy and malignant ventricular arrhythmias, as well as the challenges in managing patients in whom the offending agent cannot be safely withdrawn. Methadone prolongs ventricular repolarization primarily through blockade of the IKr channel, resulting in QT interval prolongation and increased susceptibility to early afterdepolarizations and torsades de pointes.^1^^,^^13^ The relationship between methadone dose and QT prolongation is well established, with higher doses associated with greater risk, although not strictly linear due to interindividual variability.^1^^,^^5^^,^^6^

Importantly, although torsades de pointes remains relatively uncommon, its occurrence is clinically significant due to the potential for degeneration into ventricular fibrillation and sudden cardiac death.^2^, ^3^, ^4^ In this patient, recurrent polymorphic ventricular tachycardia and ventricular fibrillation occurred despite partial dose reduction, underscoring methadone as a persistent arrhythmogenic substrate. The presence of pause-dependent initiation, short–long–short sequences, and marked QT prolongation on device electrograms further supports a triggered mechanism of arrhythmogenesis rather than primary structural or ischemic disease. Although intermittent heroin use may have contributed to at least 1 episode, the absence of heroin exposure during the most severe event supports methadone as the primary driver of arrhythmogenesis.

Although concerns may arise regarding device use in patients with limited follow-up adherence, in this case the implantable loop recorder proved critical, capturing otherwise undetected malignant ventricular arrhythmias and directly informing life-saving management. The implantable loop recorder enabled detection of both symptomatic and asymptomatic arrhythmias, including events during sleep, suggesting that arrhythmic burden may be underestimated with conventional monitoring strategies.^10^^,^^11^ In this case, device electrograms provided mechanistic insight into arrhythmia initiation and progression, which would not have been captured with intermittent monitoring modalities. Although routine loop recorder implantation is not standard in methadone-treated patients, this case raises the possibility that selected high-risk individuals—particularly those with high-dose exposure or limited follow-up—may benefit from more intensive surveillance.

A key feature of this case is the significant barrier to follow-up. Despite multiple life-threatening arrhythmic events and repeated outreach efforts, including involvement of law enforcement, the patient did not initially engage in care. Such challenges are well described in patients with substance use disorders and may contribute to delayed diagnosis and treatment of life-threatening conditions.^12^ This underscores the need for proactive and patient-centered management strategies in this population.

Finally, this case supports the role of ICD insertion for secondary prevention in patients with recurrent malignant ventricular arrhythmias, even in the absence of structural heart disease.^9^ Current American Heart Association/American College of Cardiology/Heart Rhythm Society guidelines recommend ICD therapy in patients with sustained ventricular arrhythmias or ventricular fibrillation when reversible causes cannot be fully corrected.^8^ In this patient, the inability to safely discontinue methadone, combined with recurrent polymorphic ventricular tachycardia and ventricular fibrillation with syncope, justified ICD insertion as a life-saving intervention. The overall clinical course and mechanistic insights are summarized in the Visual Summary.

## Conclusions

This case highlights methadone as a potentially potent arrhythmogenic agent capable of inducing recurrent polymorphic ventricular tachycardia and ventricular fibrillation, particularly at higher doses. When the precipitating factor cannot be safely eliminated, risk persists despite partial mitigation strategies.

Continuous rhythm monitoring may uncover clinically silent but life-threatening arrhythmias, particularly in patients with poor follow-up adherence. In such high-risk individuals, early consideration of advanced monitoring and timely intervention is critical.

For patients with recurrent malignant ventricular arrhythmias in whom risk factors are not fully reversible, ICD insertion for secondary prevention represents an appropriate and potentially life-saving strategy. This case underscores the importance of individualized care, multidisciplinary coordination, and heightened vigilance in managing arrhythmic risk in patients receiving methadone therapy.

## Funding Support and Author Disclosures

The authors have reported that they have no relationships relevant to the contents of this paper to disclose.
